# Discovery of the combined oxidative cleavage of plant xylan and cellulose by a new fungal polysaccharide monooxygenase

**DOI:** 10.1186/s13068-015-0284-1

**Published:** 2015-07-17

**Authors:** Matthias Frommhagen, Stefano Sforza, Adrie H Westphal, Jaap Visser, Sandra W A Hinz, Martijn J Koetsier, Willem J H van Berkel, Harry Gruppen, Mirjam A Kabel

**Affiliations:** 1 Laboratory of Food Chemistry, Wageningen University, Bornse Weilanden 9, 6708 WG Wageningen, The Netherlands; 2 Department of Food Science, University of Parma, Parco Area delle Scienze 59a, University Campus, 43124 Parma, Italy; 3 Laboratory of Biochemistry, Wageningen University, Wageningen, The Netherlands; 4 Dyadic Netherlands, Nieuwe Kanaal 7-S, 6709 PA Wageningen, The Netherlands

**Keywords:** Biorefinery, LPMO, Cellulose, Endoglucanase, *Myceliophthora thermophila* C1, Xylan

## Abstract

**Background:**

Many agricultural and industrial food by-products are rich in cellulose and xylan. Their enzymatic degradation into monosaccharides is seen as a basis for the production of biofuels and bio-based chemicals. Lytic polysaccharide monooxygenases (LPMOs) constitute a group of recently discovered enzymes, classified as the auxiliary activity subgroups AA9, AA10, AA11 and AA13 in the CAZy database. LPMOs cleave cellulose, chitin, starch and β-(1 → 4)-linked substituted and non-substituted glucosyl units of hemicellulose under formation of oxidized gluco-oligosaccharides.

**Results:**

Here, we demonstrate a new LPMO, obtained from *Myceliophthora thermophila* C1 (*Mt*LPMO9A). This enzyme cleaves β-(1 → 4)-xylosyl bonds in xylan under formation of oxidized xylo-oligosaccharides, while it simultaneously cleaves β-(1 → 4)-glucosyl bonds in cellulose under formation of oxidized gluco-oligosaccharides. In particular, *Mt*LPMO9A benefits from the strong interaction between low substituted linear xylan and cellulose. *Mt*LPMO9A shows a strong synergistic effect with endoglucanase I (EGI) with a 16-fold higher release of detected oligosaccharides, compared to the oligosaccharides release of *Mt*LPMO9A and EGI alone.

**Conclusion:**

Now, for the first time, we demonstrate the activity of a lytic polysaccharide monooxygenase (*Mt*LPMO9A) that shows oxidative cleavage of xylan in addition to cellulose. The ability of *Mt*LPMO9A to cleave these rigid regions provides a new paradigm in the understanding of the degradation of xylan-coated cellulose. In addition, *Mt*LPMO9A acts in strong synergism with endoglucanase I. The mode of action of *Mt*LPMO9A is considered to be important for loosening the rigid xylan–cellulose polysaccharide matrix in plant biomass, enabling increased accessibility to the matrix for hydrolytic enzymes. This discovery provides new insights into how to boost plant biomass degradation by enzyme cocktails for biorefinery applications.

**Electronic supplementary material:**

The online version of this article (doi:10.1186/s13068-015-0284-1) contains supplementary material, which is available to authorized users.

## Background

Effective degradation of plant polysaccharide biomass into monosaccharides, which are useful building blocks for biochemicals or biofuels, requires a variety of enzymes. Commercial enzyme cocktails for plant cell wall degradation mostly comprise enzymes produced by filamentous fungi, such as *Aspergillus* and *Trichoderma* strains. In addition, the commercially available fungus *Myceliophthora thermophila* C1 is a good candidate for the production of thermotolerant carbohydrate-active enzymes [1]. Still, improvements of already existing enzyme cocktails are required for a more efficient degradation of plant biomass and a decrease in costs in the production of biochemicals and biofuels.

In general, plant cell walls are composed of a primary and secondary layer, which are both built from various polymers such as polysaccharides, lignins and proteins. The polysaccharides of the plant cell wall comprise cellulose fibrils and hemicellulose [2–5]. Cellulose is a homogeneous linear polymer of β-(1 → 4)-linked glucosyl units and, depending on the source, exceeding a degree of polymerization (DP) over 10.000 [6]. These β-(1 → 4)-linked glucosyl chains form microfibrills via hydrogen bonds and van der Waals forces [7]. Depending on these interactions, cellulose is composed of crystalline and amorphous regions [8]. Lignin is an aromatic heteropolymer consisting of the three monolignols, coniferyl, sinapyl and p-coumaryl alcohol, which are methoxylated in various degrees. The network of lignin in the plant cell wall is built up of ester and ether linkages with hemicellulose [3, 9, 10]. Hemicellulose is, unlike cellulose, a very heterogeneous polymer. The hemicellulose structure differs between species of mono- and dicotyls and, depending on the source, can consist of a xylan, mannan, xyloglucan or β-glucan backbone [11–16]. The majority of these backbones are composed of β-(1 → 3, 1 → 4)-linked xylosyl, β-(1 → 4)-linked mannosyl and β-(1 → 3, 1 → 4)-linked glucosyl residues, respectively. In addition, these backbones show structural side-chain variations, which can differ in, i.e. type of substituent or distribution along the backbone [17]. Such hemicelluloses are strongly associated with cellulose via hydrogen bonding, especially the ones with a low degree of substitution or a block-wise distribution of substituents along the backbone [2, 4]. The cellulose-associated hemicelluloses block cellulases from reaching their substrate, which is likely to contribute to the defense of plants against microbial attack, and hinder the deconstruction of cellulose by industrial enzymes into fermentable monosaccharides [18, 19]. Hence, degradation of cellulose-associated hemicelluloses is essential to improve the hydrolysis of cellulose present in the plant biomass.

It has been known for a long time that enzymatic degradation of cellulosic plant biomass can be achieved by a variety of glycoside hydrolases (GHs; EC.3.2.1.), which are all listed in the Carbohydrate-Active enZYmes (CAZy, [20]) database. However, the effectiveness of hydrolases on cellulose is limited, due to hydrogen bonds between the glucan chains of the rather crystalline cellulose. Recently, it was demonstrated that the breakdown of crystalline polysaccharides could be boosted by the action of lytic polysaccharide monooxygenases (LPMOs). These LPMOs are classified as subgroups, AA9, AA10, AA11 and AA13, in the CAZy database [21]. The LPMOs exhibit oxidative cleavage between β-(1 → 4)-glucosyl units in chitin, cellulose, hemicellulosic glucan and soluble cellodextrins under formation of oxidized gluco-oligosaccharides [22–26]. Depending on the type of LPMO, the products released are gluco-oligosaccharides that are oxidized at either the reducing (C1) or the non-reducing (C4) end. Although not relevant for degradation of plant cell walls, very recently starch-active LPMO has been reported, cleaving between α-(1 → 4)-glucosyl units in starch under formation of C1-oxidized dextrins [26, 27]. This finding illustrates that LPMO activities cover a broader range of substrates than that earlier conceived. So far, no LPMO active on xylans has been reported.

In the present research, a new enzyme LPMO activity is described, which can be considered to be highly important for the concerted degradation of plant cellulose associated with xylan. In brief, we propose that the thermotolerant enzyme *Mt*LPMO9A from *M. thermophila* C1 cleaves cellulose-associated xylan under formation of oxidized xylo-oligosaccharides. In addition, to evaluate the enhanced cellulose accessibility, the synergistic effect of *Mt*LPMO9A with an endoglucanase I was determined.

## Results

### Enzyme purity

From the *M. thermophila* C1 genome, protein *Mt*LPMO9A was predicted to be an LPMO belonging to subfamily AA9 [21]. Since the addition of LPMOs to a cellulase cocktail was found to considerably increase the release of glucose from cellulose [28, 29], it was of interest to analyze the mode of action of *Mt*LPMO9A in further detail. Hereto, *Mt*LPMO9A was expressed and produced in a protease/(hemi-) cellulase-free *M. thermophila* C1 strain with Dyadic technology (Dyadic NL, Wageningen, The Netherlands). *Mt*LPMO9A was purified to apparent homogeneity using multiple chromatographic steps. The purified enzyme showed a single band in SDS-PAGE with an apparent molecular mass of 23 ± 1 kDa (Additional file 1), in good agreement with the predicted mass of *Mt*LPMO9A (22,755.3 Da; without signal peptide).

**Figure 1:**
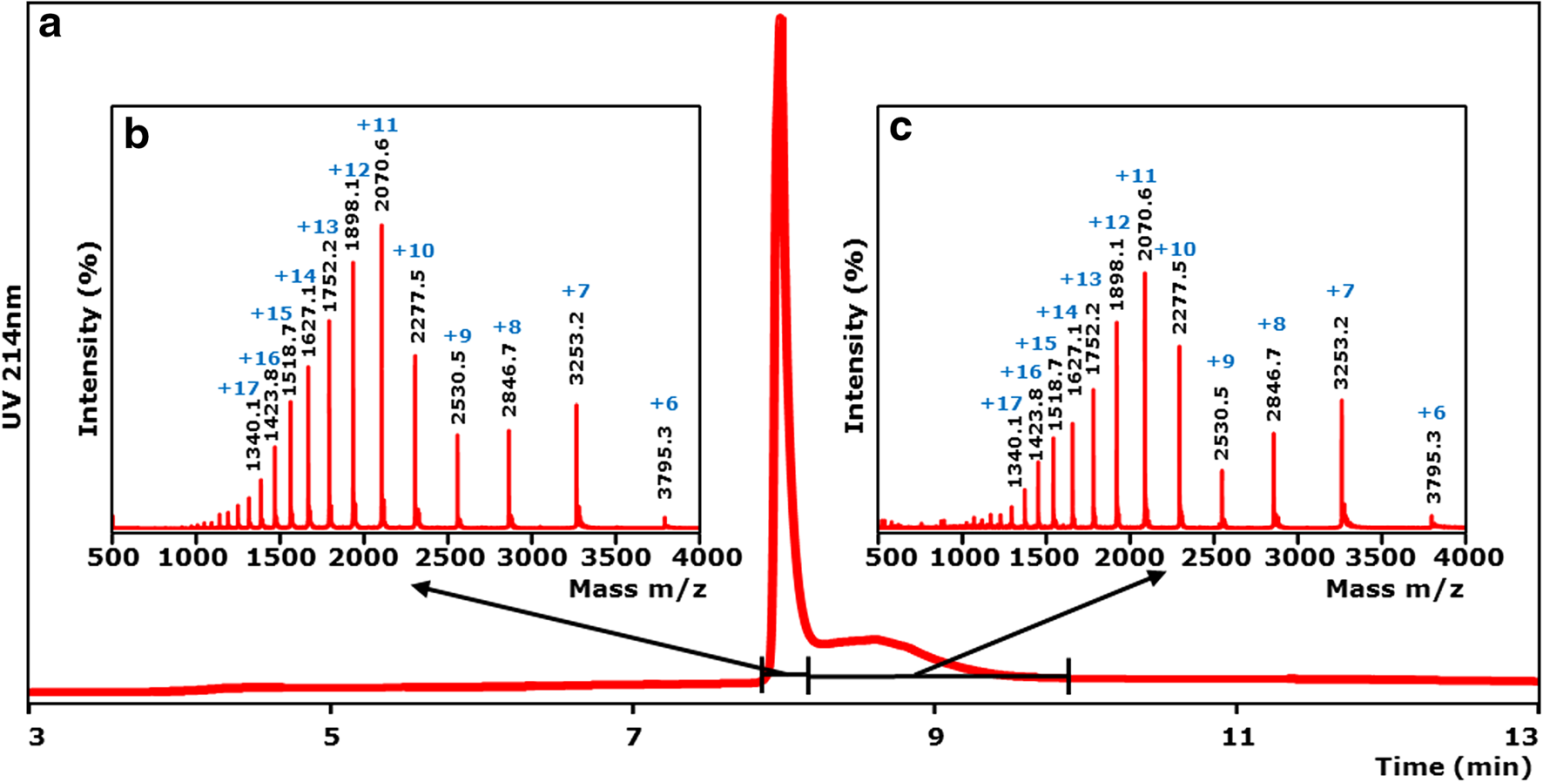
LC/ESI–MS analysis of MtLPMO9A. Purified *Mt*LPMO9A was analyzed by LC/UV/ESI-MS using an ACQUITY UPLC separation system and a SYNAPT ion mobility mass spectrometer. **a** Chromatographic profile of purified *Mt*LPMO9A (UV 214 nm). ESI MS spectra (*m*/*z* values) of the main peak (**b**) and a shallow peak (**c**) observed by UV. The main and shallow peak showed exactly the same mass spectrum corresponding to the same protein with an *m*/*z* of 22,765 Da. The main and shallow peak together featured about 99.5% of the total area measured in the UV trace at 214 nm and 94.7% of the total area measured in the total ion current (TIC) mass chromatogram (See “Methods”).* Blue numbers* represent different charge states of *Mt*LPMO9A.

To further analyze the purified *Mt*LPMO9A preparation, the enzyme was subjected to LC/UV/ESI mass spectrometry. The elution pattern (Figure 1) showed a main and a shallow peak, of which the latter was due to protein denaturation by the LC conditions applied. MS analysis of both the main and shallow peak showed exactly the same protein mass based on the multi-charged ion patterns corresponding to a mass of 22,765.3 ± 0.1 Da. The *Mt*LPMO9A protein comprised ±99.5% (65 + 34.5%, from the main and shallow peak, respectively) of the total protein present based on UV (214 nm), and 94.7% based on total ion current (TIC) in the full mass range. In conclusion, *Mt*LPMO9A was obtained in an extremely pure form.

### Structural model of *Mt*LPMO9A

**Figure 2:**
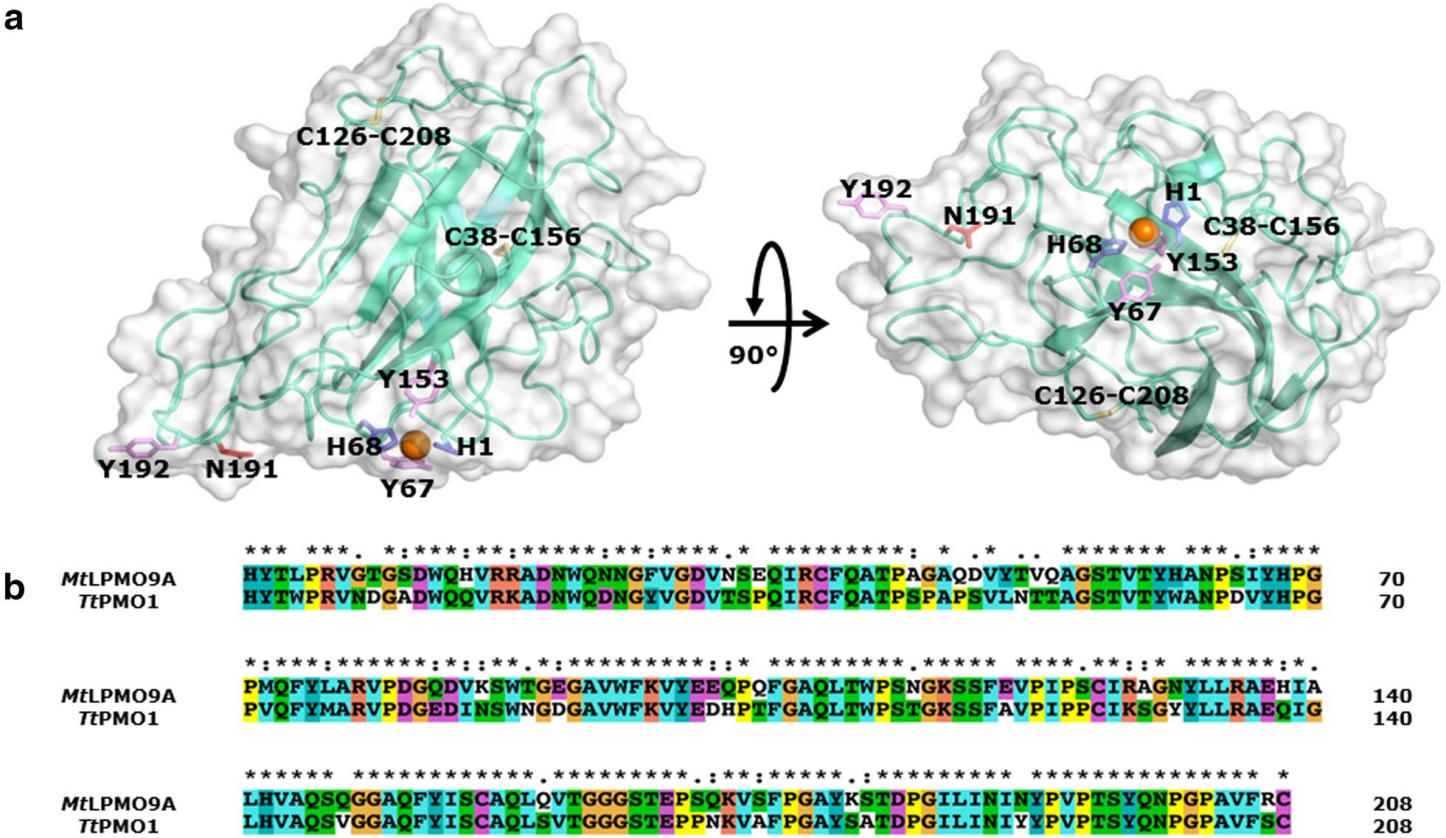
Structural model of *Mt*LPMO9A. **a** Structural model of *Mt*LPMO9A generated using the available template structure of *Tt*PMO1 from *Thielavia terrestris* (PDB-id: 3eii) [29]. The divalent metal ion (*orange*) in the flat face is coordinated by two histidines (His1 and His68; *blue*) and one tyrosine (Tyr153, *magenta*), which is typical for LPMOs belonging to subgroup AA9 of the CAZy database [30]. Compared to *Tt*PMO1, Tyr191 is replaced by Asn191 in the flat face. Two disulfide bridges, Cys126–Cys208 and Cys38–Cys156, are conserved and expected to be crucial for the thermotolerance of *Mt*LPMO9A. **b** Sequence alignment of *Mt*LPMO9A and *Tt*PMO1 (PDB-id: 3eii), which scored the highest in a Blast search using the *Mt*LPMO9A sequence against the Protein Data Bank (75% amino acid identity).

A structural model of *Mt*LPMO9A was generated based on the available structure of *Tt*PMO1 from *Thielavia terrestris* [29] (Protein Data Bank entry: 3eii), which shared 75% amino acid sequence identity. The *Mt*LPMO9A model (Figure 2) shows a highly conserved β-sheet core, whereas the loops differ from the *Tt*PMO1 structure reported. The divalent metal ion in the active site is coordinated by the three amino acids, His1, His68 and Tyr153. The tyrosine (Tyr67) above the coordination site is typical for the PMO1 subgroup of the AA9 family [30]. Of the amino acids proposed to form the flat area of the *Tt*PMO1 substrate-binding site, only one tyrosine is replaced by an asparagine in *Mt*LPMO9A (Asn191). This tyrosine is also not conserved in other LPMO structures available in the Protein Data Bank. Based on the structural model shown in Figure 2, *Mt*LPMO9A comprises two disulfide bridges, Cys126–Cys208 (−2 Da) and Cys38–Cys156 (−2 Da). The predicted mass of *Mt*LPMO9A (22,755.3 Da; amino acid sequence without signal peptide) is 10 Da lower than the actual mass determined by ESI–MS (22,765.3 Da). Based on this calculation, *Mt*LPMO9A is expected to contain a methylated N-terminal histidine (predicted mass from the amino acid sequence +14 Da for the methyl group bound to the histidine).

### Activity of *Mt*LPMO9A on amorphous cellulose

**Figure 3:**
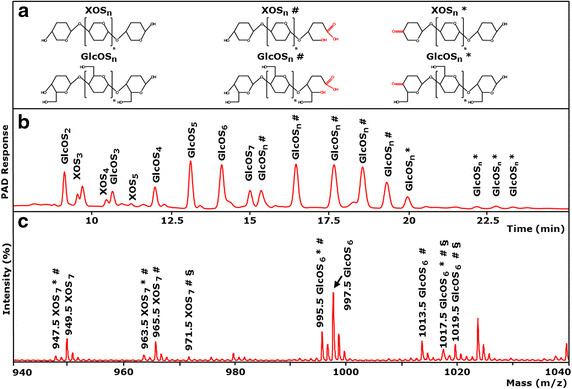
Activity of *Mt*LPMO9A on amorphous cellulose. **a** Structure and nomenclature used: XOS_n_ and GlcOS_n_, non-oxidized xylo- and gluco-oligosaccharides; XOS_n_^#^ and GlcOS_n_^#^, xylo- and gluco-oligosaccharides oxidized at the C1 carbon atom; XOS_n_* and GlcOS_n_*, xylo- and gluco-oligosaccharides oxidized at the C4 carbon atom. **b** HPAEC elution pattern of regenerated amorphous cellulose (RAC) after incubation with *Mt*LPMO9A (5 mg g^−1^ substrate). Samples were incubated in a 50 mM ammonium acetate buffer (pH 5.0) for 24 h at 52°C with ascorbic acid addition (1 mM). In the presence of ascorbic acid, oxidized GlcOS_n_^#^ * are formed by *Mt*LPMO9A (marked either with ^#^ or *), of which the masses were analyzed by MALDI-TOF MS. Using RAC as a substrate, small amounts of non-oxidized XOS_n_ are detected by HPAEC. **c** MALDI-TOF mass spectrum of RAC incubated with *Mt*LPMO9A with ascorbic acid. Clusters of oxidized GlcOS_n_^#^ * are determined as their lithium (Li) adducts. The *insert* shows masses of XOS_n_^#^ * and GlcOS_n_^#^ * oxidized either at C4 leading to a keto-group (* −2 Da) or C1 leading to a lactone (^#^ −2 Da). The δ-lactones are unstable in water and hydrolyse to the corresponding aldonic acids (^#^ +16 Da). Double Li adducts (one Li adduct and one additional Li exchanged for H on the acid group) are C1-oxidized products (^§^).

The activity of *Mt*LPMO9A was assayed on regenerated amorphous cellulose (RAC), both in the absence and presence of the external electron donor ascorbic acid. From HPAEC and MALDI-TOF MS analysis (Figure 3), it can be concluded that in the presence of ascorbic acid, RAC is degraded by *Mt*LPMO9A and that both C1 and C4 oxidized gluco-oligosaccharides (GlcOS_n_^#^ and GlcOS_n_*, respectively) and non-oxidized gluco-oligosaccharides (GlcOS_n_) were formed. Furthermore, in the absence of ascorbic acid, no oxidized and non-oxidized gluco-oligosaccharides were found, which indicates that both hydrolytic and oxidative cleavage activity toward RAC are absent (Additional file 2). The annotation by HPAEC of oxidized gluco-oligosaccharides was performed using the published elution pattern of C1- and C4-oxidized gluco-oligosaccharides formed by NCU01050 or NCU08760 from *Neurospora crassa* [22, 31]. In addition, the formation of oxidized gluco-oligosaccharides was confirmed by the masses identified with MALDI-TOF MS (Figure 3c), based on previously proposed LPMO cleaving mechanisms [31–33]. In short, oxidation at C1 of the pyranose ring leads to formation of an unstable δ-lactone, which in the presence of water converts to an aldonic acid. Lactone formation results in a 2 Da lower mass compared to the non-oxidized gluco-oligosaccharides, while aldonic acid formation results in a 16 Da higher mass (Figure 3b, c, marked with ^#^). Some of these acid groups may exchange an H ion for an Li ion, leading to a double Li adduct yielding an additional mass of 6 Da (Figure 3c, marked with ^§^). Such double adducts have also been described for galacturonic acid oligosaccharides in MALDI-TOF MS [34]. Similarly, oxidation at the C4 position leads to a 4-ketoaldose (Figure 3c, marked with *), which is rather stable. It corresponds, like the lactone, to a 2 Da lower mass compared to the non-oxidized gluco-oligosaccharides [23, 32].

Unexpectedly, besides oxidized and non-oxidized gluco-oligosaccharides, also masses of C1 and C4 oxidized xylo-oligosaccharides (XOS_n_^#^ and XOS_n_*, respectively) and non-oxidized xylo-oligosaccharides (XOS_n_) were observed (Figure 3c), although Avicel is known to contain around 2% (w/w) of xylan [35]. This striking observation suggested that *Mt*LPMO9A is capable of oxidatively cleaving xylan next to cellulose. Based on the proposed LPMO cleaving mechanism for gluco-oligosaccharides as described above, the oxidation of the C1 and C4 position of xylo-oligosaccharides leads to a 2 Da lower mass compared to non-oxidized xylo-oligosaccharides. Xylo-oligosaccharides oxidized at the C1 position are, like C1-oxidized gluco-oligosaccharides, unstable δ-lactones, which hydrolyse in water to the corresponding aldonic acids (XOS_n_^#^; +16 Da).

### Activity of *Mt*LPMO9A on three types of xylans

The observation that *Mt*LPMO9A generates oxidized xylo-oligosaccharides from RAC next to oxidized gluco-oligosaccharides is new and such an action of LPMOs has not been described for other LPMOs. Therefore, wheat arabinoxylan (WAX), birchwood glucuronoxylan (BiWX) and oat spelt xylan (OSX) were incubated with *Mt*LPMO9A in the absence or presence of ascorbic acid. The products formed were determined by using HPAEC and MALDI-TOF MS (Additional files 3, 4, 5). Remarkably, both in the absence and presence of ascorbic acid, no oxidized xylo-oligosaccharides were observed. In fact, non-oxidized xylo-oligosaccharides were released, which most likely pointed at the presence of a minor xylanolytic hydrolase impurity.

### Activity of *Mt*LPMO9A on xylan–RAC mixtures

**Figure 4:**
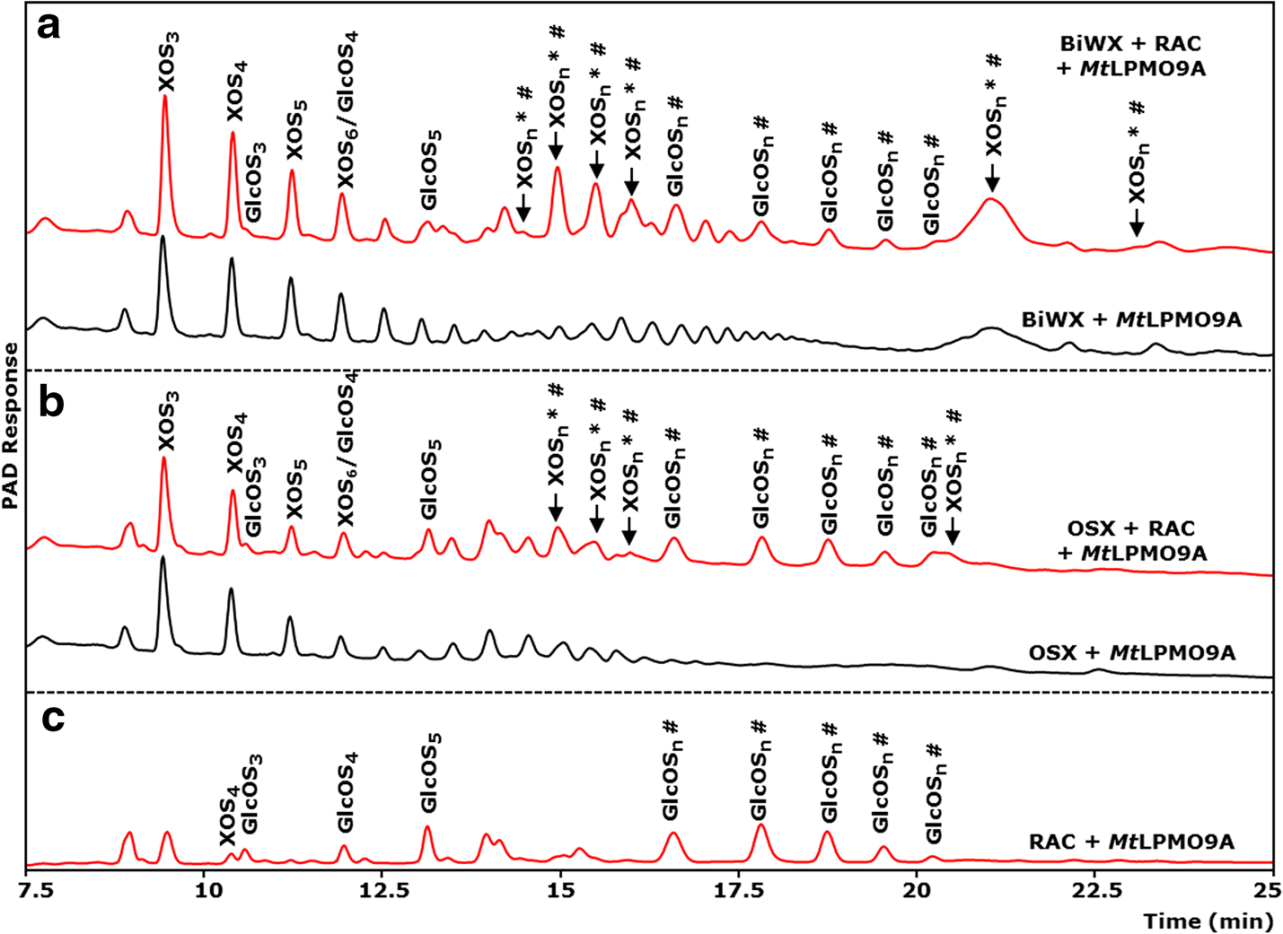
HPAEC elution pattern of xylan–RAC mixtures incubated with *Mt*LPMO9A. **a** Birchwood xylan (BiWX) and **b** oat spelt xylan (OSX) (2 mg mL^−1^) in the presence and absence of regenerated amorphous cellulose (RAC; 2 mg mL^−1^) after incubation with *Mt*LPMO9A (12.5 mg g^−1^ substrate). **c** HPAEC elution pattern of RAC after incubation with *Mt*LPMO9A (12.5 mg g^−1^ substrate). Samples were incubated in a 50 mM ammonium acetate buffer (pH 5.0) with ascorbic acid addition (1 mM). Incubation with *Mt*LPMO9A of the two xylans and xylan–RAC mixtures, in the presence of ascorbic acid, results in the formation of non-oxidized linear xylo-oligosaccharides (XOS_n_) and substituted xylo-oligosaccharides. Incubation of xylan–RAC mixtures with *Mt*LPMO9A in the presence of ascorbic acid results in the formation of non-oxidized gluco-oligosaccharides (GlcOS_n_) and oxidized gluco-oligosaccharides (GlcOS_n_^#^). The incubation of *Mt*LPMO9A with BiWX–RAC and OSX–RAC mixture in the presence of ascorbic acid results in the formation of numerous products (black arrow, indicated as oxidized xylo-oligosaccharides XOS_n_^#^ *), which are not present if *Mt*LPMO9A was incubated with BiWX, OSX or RAC alone. The results of MALDI-TOF MS analysis of BiWX–RAC and OSX–RAC mixture incubated with *Mt*LPMO9A in the presence of ascorbic acid are shown in Figure 5.

**Figure 5:**
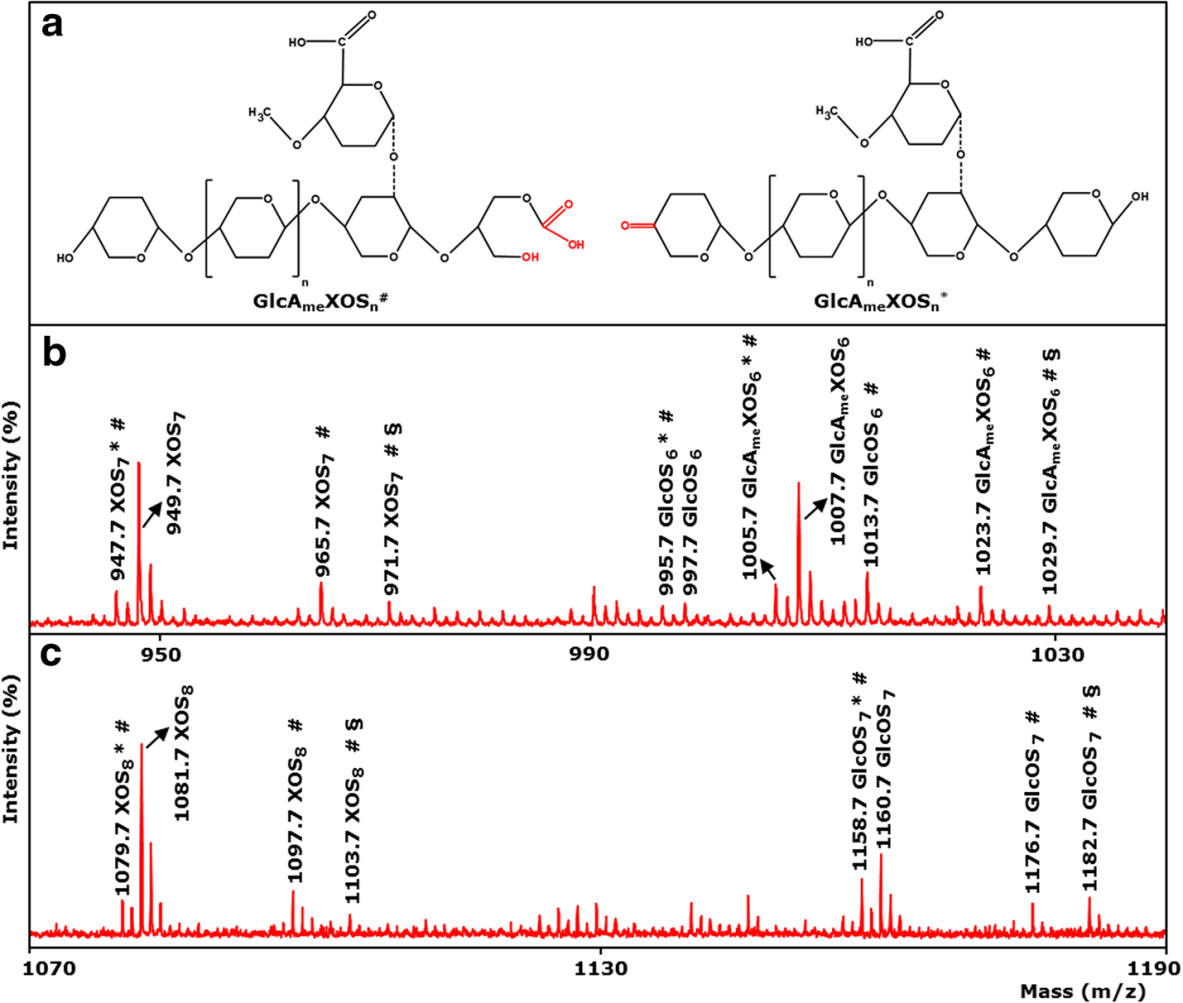
MALDI-TOF MS spectra of xylan-RAC mixtures incubated with *Mt*LPMO9A. **b** Birchwood xylan (BiWX; 2 mg mL^−1^) and **c** oat spelt xylan (OSX; 2 mg mL^−1^) in the presence of regenerated amorphous cellulose (RAC; 2 mg mL^−1^) after incubation of *Mt*LPMO9A (10 mg g^−1^ substrate). Samples were incubated in a 50 mM ammonium acetate buffer (pH 5.0) for 24 h at 52°C with ascorbic acid addition (1 mM). *Mt*LPMO9A incubation of BiWX and OSX with RAC addition releases non-oxidized and oxidized xylo- and gluco-oligosaccharides (XOS_n_, XOS_n_^#^ *; GlcOS_n_, GluOS_n_^#^ *). The presence of C4-oxidized XOS_n_*, and XOS_n_^#^ oxidized at C1 to an aldonic acid (^#^ + 16 Da) is shown. Non-oxidized GlcOS_n_ and oxidized GlcOS_n_^#^ * are less detectable due to abundance of xylo-oligosaccharides present. From BiWX also 4-*O*-methylglucoronic acid containing non-oxidized XOS_n_ (GlcA_me_XOS_n_) and 4-*O*-methylglucoronic acid containing oxidized XOS_n_^#^ * (GlcA_me_XOS_n_^#^ *) are formed. **a** Illustrated structure of 4-*O*-methylglucoronic acid containing C1- and C4-oxidized XOS_n_.(GlcA_me_XOS_n_^#^, GlcA_me_XOS_n_*, respectively). Masses represent lithium adducts only. Double Li adducts are determined for C1-oxidized products (^§^ + 6 Da). MALDI-TOF MS analysis of BiWX and OSX in the presence of RAC after incubation of *Mt*LPMO9A without ascorbic acid did not reveal detectable amounts of oxidized products (data not shown).

Since *Mt*LPMO9A generated oxidized xylo-oligosaccharides from RAC next to oxidized gluco-oligosaccharides, but not if xylan as substrate was used alone, the mode of action of *Mt*LPMO9A on xylan-rich cellulosic biomass was further investigated. Hereto, RAC was separately mixed with wheat arabinoxylan (WAX), birchwood glucuronoxylan (BiWX) or oat spelt xylan (OSX). *Mt*LPMO9A was added in the absence and presence of ascorbic acid. The products were determined using HPAEC and MALDI-TOF MS (Figures 4, 5; Additional file 5). In the absence of ascorbic acid, only non-oxidized xylo-oligosaccharides were formed from incubating RAC with BiWX, OSX or WAX. In the presence of ascorbic acid, however, the OSX–RAC and BiWX–RAC mixtures incubated with *Mt*LPMO9A showed formation of non-oxidized and oxidized xylo-oligosaccharides and of non-oxidized and oxidized GlcAme-xylo-oligosaccharides (4-*O*-methylglucuronic acid attached to xylo-oligosaccharides) next to non-oxidized and oxidized gluco-oligosaccharides (Figure 4). MALDI-TOF MS confirmed the formation of xylo-oligosaccharides and xylo-oligosaccharides oxidized at C1 (XOS_n_^#^; +16 Da) and at C4 (XOS_n_*; −2 Da). From the WAX–RAC mixture in the presence of ascorbic acid, the formation of oxidized and non-oxidized gluco-oligosaccharides was observed (Additional file 5), but not oxidized xylo-oligosaccharides.

### Synergy with EGI

**Figure 6:**
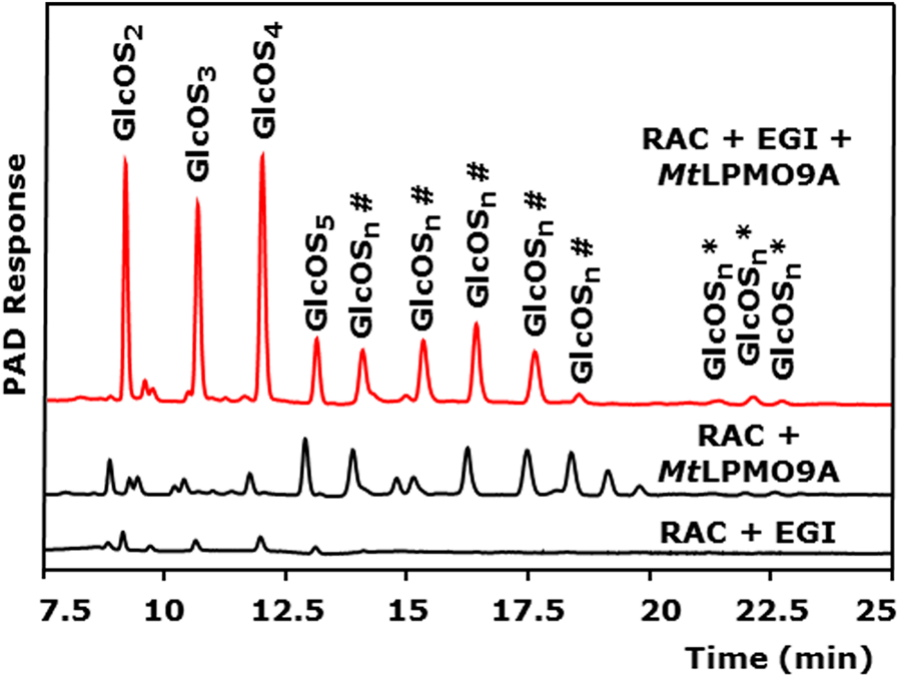
HPAEC elution patterns of RAC incubated with *Mt*LPMO9A and EGI. Regenerated amorphous cellulose (RAC, 2 mg mL^−1^) before and after incubation with *Mt*LPMO9A (10 mg g^−1^ substrate) and/or endoglucanase I from *T. viride* (EGI) (100 μg g^−1^ substrate). Samples were incubated in a 50 mM ammonium acetate buffer (pH 5.0) for 24 h at 52°C with ascorbic acid addition (1 mM). In the presence of ascorbic acid, mainly oxidized gluco-oligosaccharides (GlcOS_n_^#^ *) are formed by *Mt*LPMO9A from RAC (marked either with ^#^ for C1 or * for C4 oxidation). Incubation of EGI with RAC results in hardly detectable non-oxidized gluco-oligosaccharides (DP2-5). The combined addition of EGI and *Mt*LPMO9A results in a 16-fold higher release of non-oxidized GlcOS_n_ (based on comparison of the sum of AUC of GlcOS_2-4_ determined by HPAEC) from RAC compared to EGI incubated with RAC only.

The synergy of *Mt*LPMO9A with a pure EGI from *Trichoderma viride* [36, 37] in degrading RAC is shown in Figure 6. The release of GlcOS_n_ by EGI in the presence of *Mt*LPMO9A is around 16 and 8 times higher compared to the activity of pure EGI and pure *Mt*LPMO9A alone, respectively (based on the total HPAEC area of GlcOS_2-5_). The observed strong synergy between an LPMO and a cellulase has not been reported before.

## Discussion

LPMOs constitute a new class of oxidative enzymes, which are expected to play a crucial role in the degradation of lignocellulosic biomass [23–25, 27]. We purified a new LPMO from the commercially applied fungus *Myceliophthora thermophila* C1 and investigated its degradation capacity on a wide range of substrates (Table 1). We show for the first time an LPMO that is able to oxidize substrates with a β-(1 → 4)-linked xylan backbone, in the presence of cellulose and the electron donor ascorbic acid.

**Table 1:** *Mt*LPMO9A oxidation on various polysaccharide substrates

Substrate	Occurrence of oxidation			
	Without ascorbic acid		With 1 mM ascorbic acid	
	GlcOS_n_^#^ *^a^	XOS_n_^#^ *^b^	GlcOS_n_^#^ *	XOS_n_^#^ *
Cellulose				
Avicel^c^	−	−	+	+
RAC^c^	−	−	+	+
Hemicellulose				
Glucan				
Xyloglucan^d^	−	−	+	−
β-Glucan barley	−	−	+	−
β-Glucan oat spelt	−	−	+	−
Xylan				
OSX^e^	−	−	−	−
BiWX^e^	−	−	−	−
WAX^e^	−	−	−	−
Oligosaccharides				
Gluco-oligosaccharides^f^	−	−	−	−
Xylo-oligosaccharides^f^	−	−	−	−
Galactomannan^g^	−	−	−	−
RAC/hemicellulose combination				
RAC + BiWX	−	−	+	+
RAC + OSX	−	−	+	+
RAC + WAX	−	−	−	−

^a^Gluco-oligosaccharides oxidized at the C1 (GlcOS_n_^**#**^) or C4 position (GlcOS_n_ *).

^b^Xylo-oligosaccharides oxidized at the C1 (XOS_n_^**#**^) or C4 position (XOS_n_ *).

^c^Regenerated amorphous cellulose (RAC), crystalline cellulose (Avicel).

^d^Xyloglucan from tamarind seed.

^e^Oat spelt xylan (OSX), birchwood xylan (BiWX), wheat arabinoxylan (WAX).

^f^β-(1 → 4)-linked gluco- and xylo-oligosaccharides, degree of polymerization 2–5.

^g^β-(1 → 4)-linked-d-mannosyl backbone from guar (medium viscosity), purchased from Megazyme (Bray, Ireland).

For the formation of oxidized xylo-oligosaccharides by *Mt*LPMO9A in the presence of ascorbic acid, the addition of cellulose to xylans is essential. During incubations without ascorbic acid no oxidized xylo- and gluco-oligmers were detected. We considered the idea that the formation of glucyl radical intermediates of cellulose by *Mt*LPMO9A [31, 32] might have oxidized the xylan backbone. This hypothesis, however, seemed to be unlikely since also other LPMOs cleave cellulose under the formation of oxidized gluco-oligosaccharides via glucyl radical intermediates, but the formation of oxidized xylo-oligosaccharides has not been reported [23, 28]. We showed that the oxidized xylo-oligosaccharides detected were not from RAC only. Specifically, oxidized GlcAme-xylo-oligosaccharides from the cleavage of BiWX and OSX were also detected, but only if RAC was incubated *together* with BiWX or OSX (Figures 4, 5). Hence, we hypothesize that *Mt*LPMO9A uses the cellulose to bind while oxidizing neighboring xylan chains. This idea is strengthened by the observation that in contrast to RAC–BiWX and RAC–OSX mixtures, oxidized xylo-oligosaccharides are not formed with RAC–WAX mixtures. Unlike WAX, both BiWX and OSX consist of large sequences of unsubstituted xylosyl residues [4, 38]. Such long linear unsubstituted xylans are reported to be associated with cellulose via hydrogen bonding [2, 4]. WAX, on the other hand, has a high degree of arabinosyl substituents present on the β-(1 → 4)-linked xylan backbone [39], which has been shown to prevent association with cellulose chains [4]. Here, we show for the first time an LPMO, which benefits from the strong interaction between low substituted linear xylan and cellulose. The discovered activity of *Mt*LPMO9A provides a new paradigm in the understanding of the degradation of xylan-coated cellulose.

Recently, *Nc*LPMO9C from *N. crassa* expressed in *P. pastoris* was described to have an activity on hemicellulosic β-(1 → 4)-linked glucans [24]. We found that *Mt*LPMO9A showed a similar mode of action on hemicellulosic β-(1 → 4)-linked glucans and β-(1 → 3, 1 → 4)-linked glucans (Additional files 6, 7). However, formation of oxidized xylo-oligosaccharides so far has only been observed for *Mt*LPMO9A.

Based on their amino acids in the substrate-binding site, LPMOs of the AA9 class are further divided into the subgroups, PMOI, PMOII and PMOIII [30]. *Mt*LPMO9A shows most similarity with subgroup PMOI and has the highest amino acid sequence identity with *Tt*PMO1 (75%). Like *Tt*PMO1 [29], *Mt*LPMO9A considerably enhances glucose release from cellulose when added to a cellulase cocktail. Additionally, *Mt*LPMO9A shows a strong synergistic effect with EGI on amorphous cellulose as shown in the present study. During enzyme purification, the oxidative activity of *Mt*LPMO9A was separated from a strong hydrolytic activity toward cellulose (data not shown). Probably, *Mt*LPMO9A and the enzyme responsible for this hydrolytic activity closely work together in vivo (Additional file 8). Possibly, during the evolution of fungi, the development of enzymes containing both oxidative activities and synergism with hydrolases enabled a more efficient degradation of a wider range of substrates present in nature.

## Conclusion

The enzymatic degradation of cellulose and xylan-rich agricultural and industrial food by-products into monosaccharides is seen as a basis for the production of biofuels and bio-based chemicals. Now, for the first time, we demonstrate the activity of a lytic polysaccharide monooxygenase (*Mt*LPMO9A) that shows oxidative cleavage of xylan in addition to cellulose and that acts in synergism with endoglucanase I. The ability of *Mt*LPMO9A to cleave the xylan-coated cellulose regions is considered to be important for loosening the rigid plant polysaccharide matrix in plant biomass, enabling an increased accessibility for hydrolytic enzymes. This discovery provides new insights into how fungi degrade plant cell wall structures by using both oxidative activity and synergism with hydrolases and, in addition, how to boost hydrolytic enzyme cocktails for biorefinery applications.

## Methods

### Enzyme expression, production and purification

*Mt*LPMO9A from *Myceliophthora thermomphila* C1 (UniProt: KP901251) was over-expressed in a protease/(hemi-) cellulase-free C1-expression host (LC strain) [40, 41]. The C1 strain was grown aerobically in 2-L fermentors using a medium containing glucose and ammonium sulfate, and enriched with essential salts [41]. Enzyme production was performed under glucose limitation in a fed-batch process (pH 6.0; 32°C) as described previously [40] and resulted in an *Mt*LPMO9A-rich crude enzyme extract. The crude enzyme extract was dialyzed against 10 mM potassium phosphate buffer (pH 7.0). *Mt*LPMO9A was purified using an AKTA-Explorer preparative chromatography system (GE Healthcare, Uppsala, Sweden). As a first step, 3 g of the dialyzed crude enzyme mixture (50 mg mL^−1^) was subjected to a self-packed Source 15Q column (100 × 70 mm internal diameter, GE Healthcare), pre-equilibrated in 20 mM potassium phosphate buffer (pH 7.0). After protein application, the column was washed with three column volumes of 20 mM potassium phosphate buffer (pH 7.0). Elution was performed with a linear gradient of 0–1 M NaCl in 20 mM potassium phosphate buffer (pH 7.0) over five column volumes at 25 mL min^−1^. The eluate was monitored at 220 and 280 nm. Fractions (20 mL) were collected and immediately stored on ice. Peak fractions were pooled and concentrated using ultrafiltration (Amicon Ultra, molecular mass cut-off of 3 kDa, Merck Millipore, Cork, Ireland) at 4°C. The concentrated pools were subjected to SDS-PAGE (Additional file 1). For further purification (2nd step), the *Mt*LPMO9A-containing pool (fraction AEC-I, Additional file 1) was loaded onto a self-packed Superdex TM-75 column (100 × 3 cm internal diameter, GE Healthcare) and eluted at 5 mL min^−1^ with a 10 mM potassium phosphate buffer (pH 7.0) containing 150 mM NaCl. Fractions (5 mL) were immediately stored on ice. Peak fractions were pooled and concentrated by ultrafiltration as described above.

The *Mt*LPMO9A preparation thus obtained (partially purified fraction SEC-I; Additional file 1) was further subjected (3rd step) to a Resource Q column (30 × 16 mm internal diameter, GE Healthcare), pre-equilibrated in 20 mM potassium phosphate buffer (pH 7.0). After protein application, the column was washed with 20 column volumes of starting buffer. Elution at 6 mL min^−1^ was performed with a linear gradient of 0–1 M NaCl in 20 mM potassium phosphate buffer (pH 7.0) over 20 column volumes. Elution was monitored at 220 and 280 nm. Fractions (3 mL) were immediately stored on ice. Peak fractions were pooled and concentrated by ultrafiltration as described above.

### Protein identification

Sequencing of the *Mt*LPMO9A coding sequence was carried out by the Scripps Research Institute, USA.

### Protein content

To analyze protein contents, the BCA Protein Assay Kit (Thermo Scientific, Rockford, IL, USA) was used with bovine serum albumin (BSA) as calibration.

### SDS-PAGE

The protein purity was analyzed by using sodium dodecyl sulfate-polyacrylamide gel electrophoresis (SDS-PAGE). Therefore, proteins were reduced with β-mercaptoethanol, heated for 10 min and loaded on 12% polyacrylamide gels (Mini-PROTEAN TGX Gels, Bio-Rad Laboratories, Hempel Hempstead, UK). In addition, a protein marker (Protein All Blue Standards, Bio-Rad Laboratories) was loaded for mass calibration. Gels were stained with the EZBlue Gel Staining Reagent (Sigma Aldrich, Steinheim, Germany).

### LC/ESI–MS

Purified *Mt*LPMO9A (2.5 mg mL^−1^ in 0.1% (v/v) trifluoroacetic acid (TFA) in H_2_O) was analyzed by liquid chromatography/electron spray ionization-mass spectrometry (LC/ESI–MS) using an ACQUITY UPLC separation system (Waters, Milford, MA, USA) equipped with a C4-reversed phase column (UPLC BEH C4 1.7μm, 2.1 × 100 mm, Waters) coupled to a PLC LG 500 photodiode array detector (Waters) and a SYNAPT G2-Si High Definition Mass Spectrometer (Waters). Gradient elution with eluent A (H_2_O + 1% (v/v) acetonitrile + 0.1% (v/v) TFA) and eluent B (acetonitrile + 0.1% (v/v) TFA) was performed according to the following steps: From 0 to 2 min isocratic 90% A, from 2 to 12 min gradient from 90% A to 25% A, from 12 to 15 min gradient from 25% A to 100% B and from 12 to 15 min isocratic at 100% B; then re-equilibration to the initial conditions. The flow rate and the injection volume were 0.35 mL min^−1^ and 2 μL, respectively. The photodiode array detector was operated at a sampling rate of 40 points sec^−1^ in the range 200–400 nm, resolution 1.2 nm. The SYNAPT mass spectrometer was operated in the positive ion mode (resolution mode), capillary voltage 3 kV, sampling cone 30 V, source temperature 150°C, desolvation temperature 500°C, cone gas flow (N_2_) 200 L h^−1^, desolvation gas flow (N_2_) 800 L h^−1^, acquisition in the full scan mode, scan time 0.3 s, interscan time 0.015 s, acquisition range 150–4,000 *m*/*z*.

### Substrates incubated with *Mt*LPMO9A

OSX, BiWX, Avicel PH-101, xylo-oligosaccharides (DP1-5) and β-(1 → 4)-linked gluco-oligosaccharides (DP1-5) were obtained from Sigma-Aldrich (Steinheim, Germany). WAX (medium viscosity), β-(1 → 3, 1 → 4)-linked glucan from barley (medium viscosity) and oat spelt (medium viscosity) were purchased from Megazyme (Bray, Ireland). Xyloglucan (XG; from tamarind seed) was obtained from Dainippon Sumitomo Pharma (Osaka, Japan). Regenerated amorphous cellulose (RAC) was prepared from Avicel PH-101 by adopting a method described elsewhere [42]. Briefly, Avicel PH-101 (100 mg) was moistened with 0.6 mL water. Next, 10 mL 86.2% (w/v) *ortho*-phosphoric acid was slowly added followed by rigorous stirring for 30 min until the cellulose was completely dissolved. The dissolved cellulose precipitated during stepwise addition of 40 mL of water. After centrifugation (4,000*g*, 12 min, 4°C), the pellet obtained was washed twice with water and neutralized (pH 7.0) with 2 M sodium carbonate. The pellet was washed again with water (three times) and the final pellet was suspended in water to a dry matter content of 1.4 ± 0.1% (w/w) RAC suspension.

### *Mt*LPMO9A activity assays

Substrates (1–2 mg mL^−1^, see Figure captions) were dissolved in 50 mM ammonium acetate buffer (pH 5.0), with or without addition of ascorbic acid (final concentration of 1 mM). *Mt*LPMO9A was added (12.5 µg mg^−1^ substrate) and incubated for 24 h at 50°C in a head-over-tail rotator in portions of 1 mL total volume (Stuart rotator, Bibby Scientific, Stone, UK) at 20 rpm. Supernatants of all incubations, including substrates incubated with and without ascorbic acid in the absence of *Mt*LPMO9A, were analyzed by HPAEC and MALDI-TOF MS.

### Structural modelling

An alignment was made of the amino acid sequence of *Mt*LPMO9A and the amino acid sequence of PMO1 from *Thielavia terrestris*, which scored highest in a Blast search using the *Mt*LPMO9A sequence against the Protein Data Bank (75% amino acid identity). Using this alignment and the available structure of *Tt*PMO1 (PDB-id: 3eii) as template, structural models were obtained for *Mt*LPMO9A using the Modeller program version 9.14 [43]. Thirty comparative models were generated, after which the model with the lowest corresponding DOPE score [44] was selected for image generation using Pymol (Pymol, The PyMOL Molecular Graphics System, Version 1.5.0.4 Schrödinger, LLC, New York, NY, USA).

### Oligosaccharides analysis

Oligosaccharides were analyzed by high-performance anion exchange chromatography (HPAEC) with pulsed amperometric detection (PAD). The HPAEC system (ICS-5000, Dionex, Sunnyvale, CA, USA) was equipped with a combination of a CarboPac PA1 guard column (50 mm × 2 mm i.d., Dionex) and a CarboPac PA1 analytical column (250 mm × 2 mm i.d., Dionex). The flow rate was 0.3 mL min^−1^ (20°C). Samples were kept at 6°C in the autosampler and the injection volume was 10 µL. Elution was performed using two mobile phases: 0.1 M NaOH and 1 M NaOAc in 0.1 M NaOH. The gradient elution program was as follows: 0–30 min, linear gradient 0–400 mM NaOAc; 30–40 min linear gradient 400–1,000 mM NaOAc; followed by a cleaning step and equilibration (15 min) of the column with the starting conditions. Soluble gluco- and xylo-oligosaccharides (degree of polymerization 1–5) as well as glucuronic and gluconic acid were used as standards (Sigma-Aldrich).

### MALDI-TOF MS

For matrix-assisted laser desorption ionization-time of flight mass spectrometry (MALDI-TOF MS), an Ultraflex workstation using FlexControl 3.3 (Bruker Daltonics) equipped with a nitrogen laser of 337 nm was used. The pulsed ion extraction was set on 80 ns. Ions were accelerated to a kinetic energy of 25 kV and detected in positive reflector mode with a set reflector voltage of 26 kV. The lowest laser energy required was used to obtain a good signal-to-noise ratio. A total of 200 spectra were collected for each measurement. The mass spectrometer was calibrated using a mixture of maltodextrins (Avebe, Veendam, The Netherlands) in a mass range (*m*/*z*) of 500–2,500. The peak spectra were processed by using FlexAnalysis software version 3.3 (Bruker Daltonics). Prior to analysis, samples were desalted by adding AG 50 W-X8 Resin (Bio-Rad Laboratories). To obtain lithium (Li) adducts, the supernatant was dried under nitrogen and re-suspended in 20 mM LiCl [28]. Each lithium-enriched sample of a volume of 1 µL was mixed with 1 µL of matrix solution (12 mg mL^−1^ 2,5-dihydroxy-benzoic acid (Bruker Daltonics) in 30% (v/v) acetonitrile in H_2_O), applied on an MTP 384 massive target plate (Bruker Daltonics) and dried under a stream of warm air.

### Additional files

Additional file 1: **Figure 1.** The purification of LPMO9A from *Myceliophthora thermophila C1.* A – Anion exchange chromatography (AEC) elution profile (step 1) of crude enzyme extract containing expressed *Mt*LPMO9A. B – Size exclusion chromatography (SEC) elution profile of Pool AEC-I (step 2). The framed columns indicate the *Mt*LPMO9A-containing fractions pooled and concentrated for further analysis. C - SDS-PAGE of marker (lane 1; Precision Plus Protein, Bio-Rad Laboratories), the crude enzyme extract (lane 2), pooled fraction AEC-I (lane 3) and partially purified fraction SEC-I (lane 4). Protein bands corresponding to *Mt*LPMO9A are indicated by an arrow. For more details about protein purification see Materials and Methods.

Additional file 2: **Figure 2.** The HPAEC elution patterns and MALDI-TOF mass spectrum of cellulose incubated with *Mt*LPMO9A. A - Regenerated amorphous cellulose (RAC; 2mg mL^-1^) before and after incubation with *Mt*LPMO9A (12.5 mg g^-1^ substrate). Samples were incubated in a 50 mM ammonium acetate buffer (pH 5.0) for 24 h at 52°C, either with ascorbic acid addition (1 mM) or without. In the presence of *Mt*LPMO9A and ascorbic acid, non-oxidized gluco-oligomers (GlcOS_n_) and gluco-oligomers oxidized at C1 (GlcOS_n_^#^) and C4 (GlcOS_n_*) are formed from RAC. Neither non-oxidized nor oxidized gluco-oligomers were formed by *Mt*LPMO9A in the absence of ascorbic acid. In both incubations of RAC with *Mt*LPMO9A, either with or without ascorbic acid, traces of non-oxidized xylo-oligomers were formed (XOS_n_). B - MALDI-TOF mass spectrum of RAC incubated with *Mt*LPMO9A with ascorbic acid. Clusters of oxidized gluco-oligomers are determined as their lithium (Li) adducts. See Figure 3 for more details.

Additional file 3: **Figure 3.** The MALDI-TOF MS analysis of xylan incubated with *Mt*LPMO9A. (A) Wheat arabinoxylan (WAX), (B) birchwood (BiWX) and (C) oat spelt (OSX) xylan (2mg mL^-1^) after incubation with *Mt*LPMO9A (12.5 mg g^-1^ substrate). Samples were incubated in a 50 mM ammonium acetate buffer (pH 5.0) containing ascorbic acid addition (1 mM) for 24 h at 52°C. In all three incubations, *Mt*LPMO9A released non-oxidized xylo-oligomers (XOS_n_). A - incubation of WAX with *Mt*LPMO9A; formation of non-oxidized xylo-oligomers and traces of acetylated (_acetyl_XOS_n_) xylo-oligomers (+42 Da). B - incubation of BiWX with *Mt*LPMO9A; formation of non-oxidized xylo-oligomers and xylo-oligomers (Glc_me_XOS_n_) substituted with 4-O-methyl-glucoronic acid (+191 Da). C - incubation of OSX with *Mt*LPMO9A releases non-oxidized xylo-oligomers only. Masses represents lithium (Li) adducts only.

Additional file 4: **Figure 4.** The HPAEC elution patterns of xylan incubated with *Mt*LPMO9A. Birchwood xylan (BiWX), oat spelt xylan (OSX) and wheat arabinoxylan (WAX) (2 mg mL^-1^) before and after incubation with *Mt*LPMO9A (12.5 mg g^-1^ substrate). Samples were incubated in a 50 mM ammonium acetate buffer (pH 5.0) containing ascorbic acid addition (1 mM) for 24 h at 52°C. Incubation with *Mt*LPMO9A results in the formation of non-oxidized linear xylo-oligomers (XOS_n_) and substituted xylo-oligomers (black dashed arrow).

Additional file 5: **Figure 5.** The HPAEC elution patterns of *Mt*LPMO9A incubations with xylan and xylan-RAC mixtures. (A) birchwood xylan (BiWX), (B) oat spelt xylan (OSX) and (C) wheat arabinoxylan (WAX) (2 mg mL^-1^) in the presence and absence of regenerated amorphous cellulose (RAC; 2 mg mL^-1^) before and after incubation with *Mt*LPMO9A (12.5 mg g^-1^ substrate). Samples were incubated in a 50 mM ammonium acetate buffer (pH 5.0) with ascorbic acid addition (1 mM) or without for 24 h at 52°C. Incubation with *Mt*LPMO9A of all three xylans and xylan-RAC mixtures, in the presence or absence of ascorbic acid, results in the formation of non-oxidized linear xylo-oligomers (XOS_n_) and substituted xylo-oligomers (black dashed arrow). Incubation of xylan-RAC mixtures with *Mt*LPMO9A in the presence of ascorbic acid results in the formation of non-oxidized gluco-oligomers (GlcOS_n_) and C1-oxidized gluco-oligomers (GlcOS_n_^#^). A + B – The incubation of *Mt*LPMO9A with BiWX-RAC and OSX-RAC mixture in the presence of ascorbic acid results in the formation of numerous products (black arrow), which are not present if *Mt*LPMO9A is incubated with BiWX, OSX or RAC alone. The results of MALDI-TOF MS analysis of BiWX-RAC and OSX-RAC mixture incubated with *Mt*LPMO9A in the presence of ascorbic acid are shown in Figure 4.

Additional file 6: **Figure 6.** The HPAEC and MALDI-TOF MS analysis of oat spelt (OS) β-glucan incubated with *Mt*LPMO9A. A - HPAEC elution pattern OS β-glucan before and after incubation with partially purified *Mt*LPMO9A fraction SEC-I (Additional Figure 1; 12.5 mg g^-1^ substrate), with addition of 1 mM ascorbic acid or without. A - In the presence and absence of ascorbic acid, various non-oxidized β-gluco-oligomers (GlcOS_n_) are formed by the partially purified *Mt*LPMO9A fraction. Incubation of oat spelt β-glucan with *Mt*LPMO9A in the presence of ascorbic acid results in the formation of oxidized gluco-oligomers (GlcOS_n_^#^, GlcOS_n_*). B – MALDI-TOF mass spectrum of partially purified *Mt*LPMO9A incubated with oat spelt β-glucan in the presence of 1 mM ascorbic acid. Clusters of non-oxidized gluco-oligomers and gluco-oligomers, oxidized at C1 (GlcOS_n_^#^) and C4 (GlcOS_n_*) are determined. Enlargement (C) shows the presence of non-oxidized gluco-oligomers and gluco-oligomers, oxidized at C1 with an aldonic acid (^#^) and at C4 with a keto-group (*). Masses represent lithium (Li) adducts only.

Additional file 7: **Figure 7.** The HPAEC and MALDI-TOF MS analysis of xyloglucan incubated with *Mt*LPMO9A. A - HPAEC elution pattern of xyloglucan from tamarind seed (2 mg mL^-1^) after incubation with partially purified *Mt*LPMO9A fraction SEC-I (Additional Figure 1; 12.5 mg g^-1^ substrate). Samples were incubated in 50 mM ammonium acetate buffer (pH 5.0) for 24h at 52°C, either with ascorbic acid addition (1 mM) or without. Numerous various non-oxidized xyloglucan-derived oligomers were formed if xyloglucan was incubated with *Mt*LPMO9A, either with ascorbic acid addition (1 mM) or without. B - MALDI-TOF mass spectrum of xyloglucan incubated with *Mt*LPMO9A with 1 mM ascorbic acid addition. Clusters of non-oxidized and oxidized xyloglucan-derived oligomers are formed. C (enlargement of B) - In the presence of *Mt*LPMO9A and ascorbic acid, next to non-oxidized xyloglucan-derived oligomers, oligomers oxidized at the C1 (^#^) and C4 (*) position are formed. Masses represent lithium (Li) adducts only.

Additional file 8: **Figure 8.** *Mt*LPMO9A incubation with a cellulase cocktail. HPAEC elution patterns of Avicel incubations with a cellulase cocktail (Dyadic, Wageningen, The Nederlands) with and without partially purified *Mt*LPMO9A addition (2.5 mg protein g^-1^ Avicel). The addition of *Mt*LPMO9A to a cellulase cocktail (5 mg protein g^-1^ Avicel) results in a 60% higher release of glucose (based on HPAEC-area) compared to the glucose release from Avicel by the cellulase cocktail alone. Samples were incubated in 50 mM acetate buffer (pH 5.0) at 52°C with ascorbic acid addition (1 mM).

## Abbreviations

AA: auxiliary activity
BiWX: birchwood glucuronoxylan
CAZy: carbohydrate-active enzymes
EGI: endoglucanase I
GH: glycoside hydrolases
GlcAmeXOS_n_^#^: 4-*O*-methylglucoronic acid containing C1-oxidized xylo-oligosaccharides
GlcAmeXOS_n_*: 4-*O*-methylglucoronic acid containing C4-oxidized xylo-oligosaccharides
GlcAmeXOS_n_: 4-*O*-methylglucoronic acid containing non-oxidized xylo-oligosaccharides
GlcOS_n_^#^: C1-oxidized gluco-oligosaccharides
GlcOS_n_*: C4 oxidized gluco-oligosaccharides
GlcOS_n_: non-oxidized gluco-oligosaccharides
HPAEC: high performance anion exchange chromatography
LC/ESI–MS: liquid chromatography/electronspray ionization-mass spectrometry
LPMO: lytic polysaccharide monooxygenases
MALDI-TOF MS: matrix-assisted laser desorption ionization-time of flight mass spectrometry
OSX: oat spelt xylan
PAD: pulsed amperometric detection
RAC: regenerated amorphous cellulose
SDS-PAGE: sodium dodecyl sulfate-polyacrylamide gel electrophoresis
TIC: total ion current
WAX: wheat arabinoxylan
XOS_n_^#^: C1-oxidized xylo-oligosaccharides
XOS_n_*: C4-oxidized xylo-oligosaccharides
XOS_n_: non-oxidized xylo-oligosaccharides
