# Supplementary figures and images for: Discovery of the combined oxidative cleavage of plant xylan and cellulose by a new fungal polysaccharide monooxygenase
Source: Biotechnol Biofuels. 2015 Jul 17;8:101. doi: 10.1186/s13068-015-0284-1 (PMC4504452; doi:10.1186/s13068-015-0284-1)

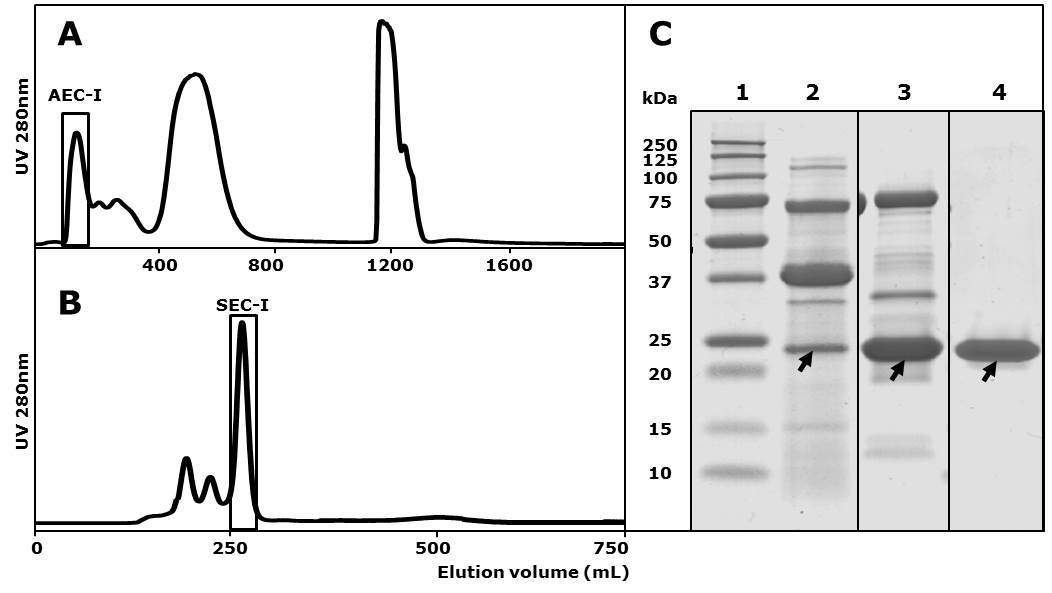

Supplement: Supplementary file 1 — Figure 1. The purification of LPMO9A from Myceliophthora thermophila C1. A – Anion exchange chromatography (AEC) elution profile (step 1) of crude enzyme extract containing expressed MtLPMO9A. B – Size exclusion chromatography (SEC) elution profile of Pool AEC-I (step 2). The framed columns indicate the MtLPMO9A-containing fractions pooled and concentrated for further analysis. C - SDS-PAGE of marker (lane 1; Precision Plus Protein, Bio-Rad Laboratories), the crude enzyme extract (lane 2), pooled fraction AEC-I (lane 3) and partially purified fraction SEC-I (lane 4). Protein bands corresponding to MtLPMO9A are indicated by an arrow. For more details about protein purification see Materials and Methods. [file 13068_2015_284_MOESM1_ESM.jpg]

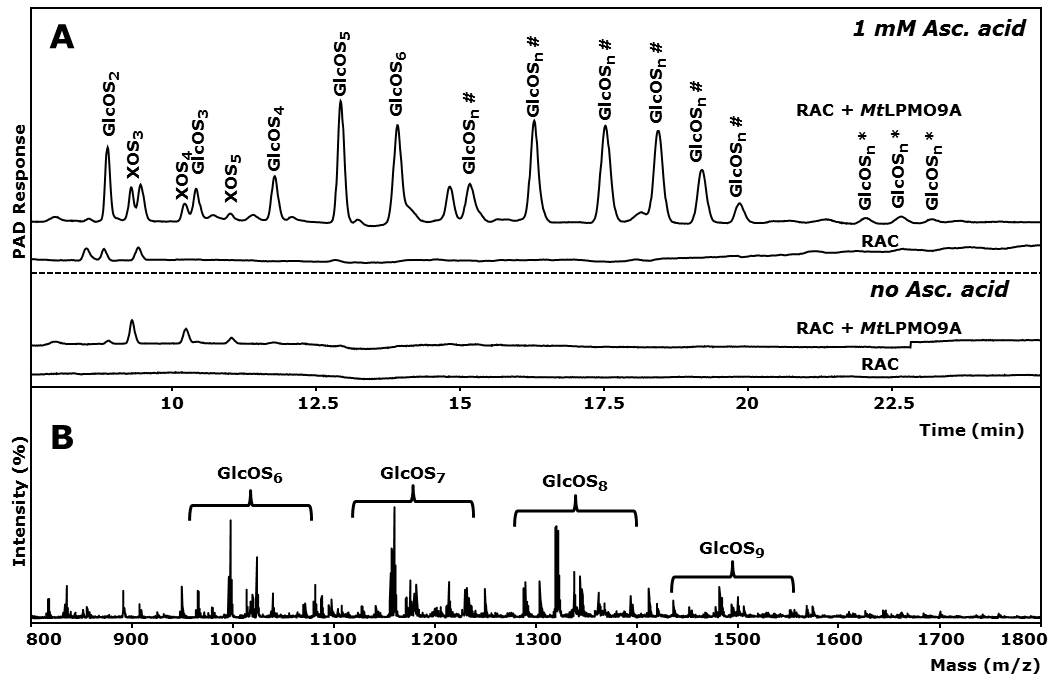

Supplement: Supplementary file 2 — Figure 2. The HPAEC elution patterns and MALDI-TOF mass spectrum of cellulose incubated with MtLPMO9A. A - Regenerated amorphous cellulose (RAC; 2mg mL-1) before and after incubation with MtLPMO9A (12.5 mg g-1 substrate). Samples were incubated in a 50 mM ammonium acetate buffer (pH 5.0) for 24 h at 52°C, either with ascorbic acid addition (1 mM) or without. In the presence of MtLPMO9A and ascorbic acid, non-oxidized gluco-oligomers (GlcOSn) and gluco-oligomers oxidized at C1 (GlcOSn#) and C4 (GlcOSn*) are formed from RAC. Neither non-oxidized nor oxidized gluco-oligomers were formed by MtLPMO9A in the absence of ascorbic acid. In both incubations of RAC with MtLPMO9A, either with or without ascorbic acid, traces of non-oxidized xylo-oligomers were formed (XOSn). B - MALDI-TOF mass spectrum of RAC incubated with MtLPMO9A with ascorbic acid. Clusters of oxidized gluco-oligomers are determined as their lithium (Li) adducts. See Figure 3 for more details. [file 13068_2015_284_MOESM2_ESM.jpg]

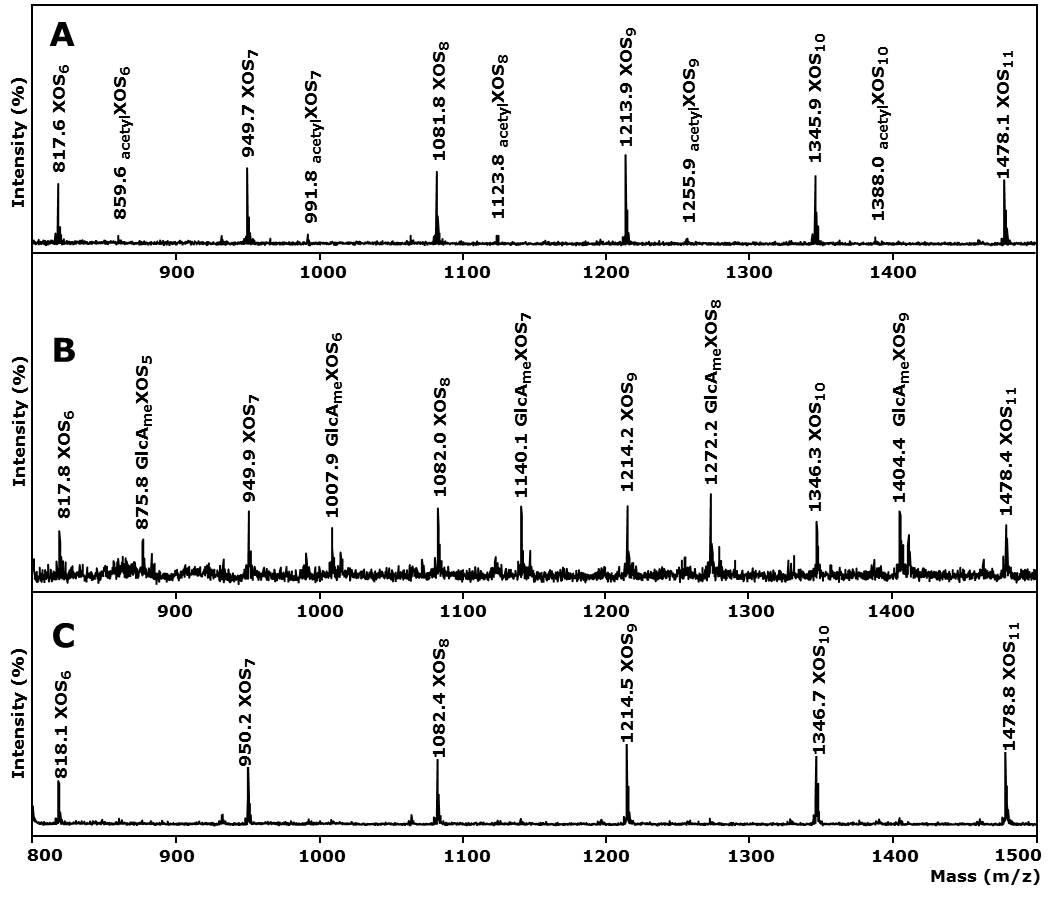

Supplement: Supplementary file 3 — Figure 3. The MALDI-TOF MS analysis of xylan incubated with MtLPMO9A. (A) Wheat arabinoxylan (WAX), (B) birchwood (BiWX) and (C) oat spelt (OSX) xylan (2mg mL-1) after incubation with MtLPMO9A (12.5 mg g-1 substrate). Samples were incubated in a 50 mM ammonium acetate buffer (pH 5.0) containing ascorbic acid addition (1 mM) for 24 h at 52°C. In all three incubations, MtLPMO9A released non-oxidized xylo-oligomers (XOSn). A - incubation of WAX with MtLPMO9A; formation of non-oxidized xylo-oligomers and traces of acetylated (acetylXOSn) xylo-oligomers (+42 Da). B - incubation of BiWX with MtLPMO9A; formation of non-oxidized xylo-oligomers and xylo-oligomers (GlcmeXOSn) substituted with 4-O-methyl-glucoronic acid (+191 Da). C - incubation of OSX with MtLPMO9A releases non-oxidized xylo-oligomers only. Masses represents lithium (Li) adducts only. [file 13068_2015_284_MOESM3_ESM.jpg]

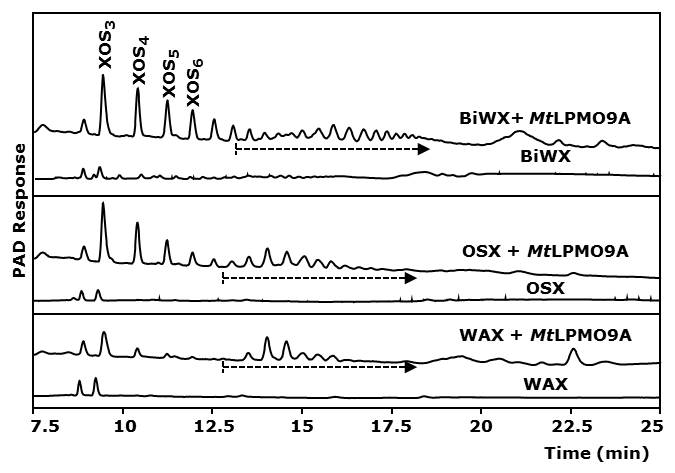

Supplement: Supplementary file 4 — Figure 4. The HPAEC elution patterns of xylan incubated with MtLPMO9A. Birchwood xylan (BiWX), oat spelt xylan (OSX) and wheat arabinoxylan (WAX) (2 mg mL-1) before and after incubation with MtLPMO9A (12.5 mg g-1 substrate). Samples were incubated in a 50 mM ammonium acetate buffer (pH 5.0) containing ascorbic acid addition (1 mM) for 24 h at 52°C. Incubation with MtLPMO9A results in the formation of non-oxidized linear xylo-oligomers (XOSn) and substituted xylo-oligomers (black dashed arrow). [file 13068_2015_284_MOESM4_ESM.jpg]

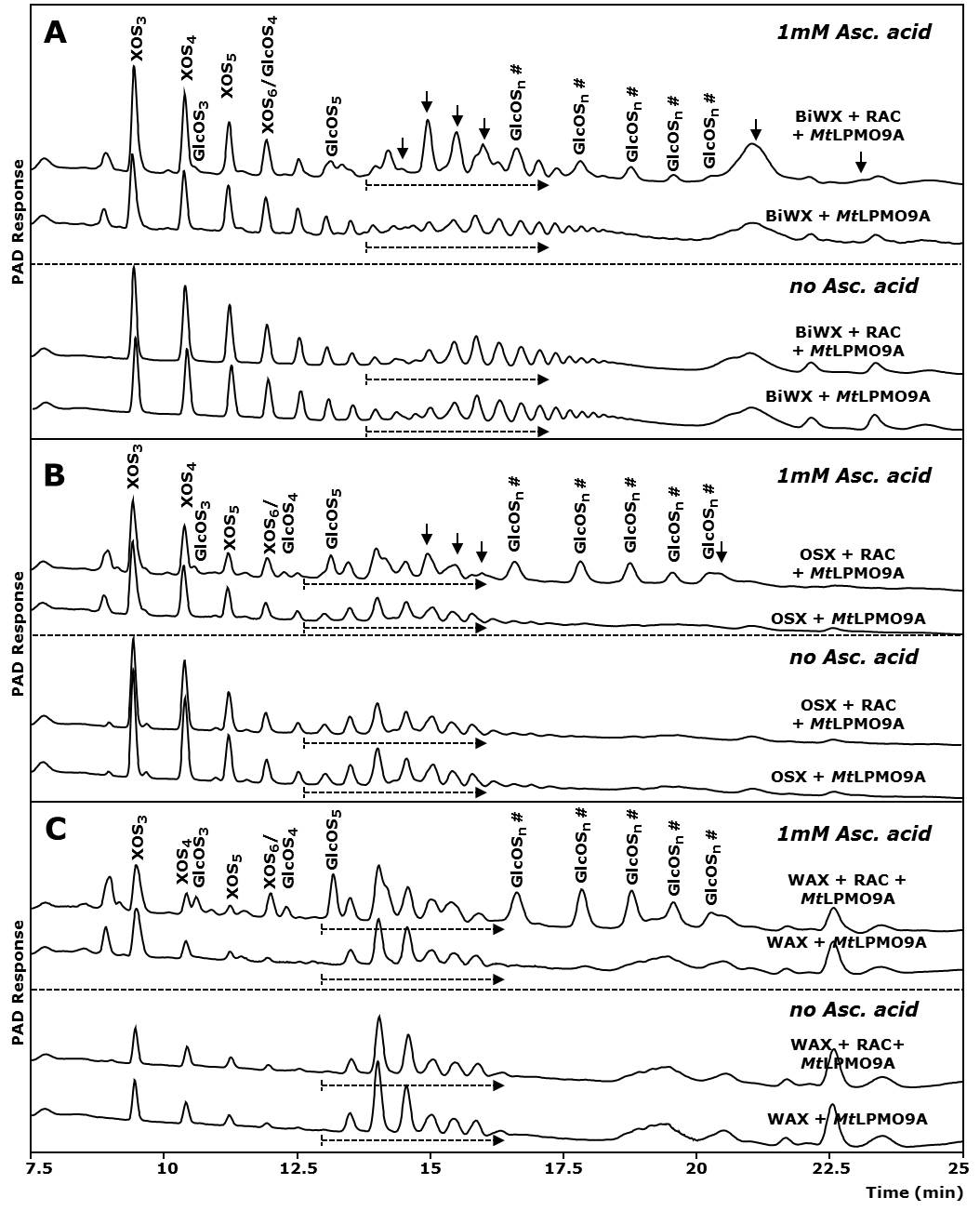

Supplement: Supplementary file 5 — Figure 5. The HPAEC elution patterns of MtLPMO9A incubations with xylan and xylan-RAC mixtures. (A) birchwood xylan (BiWX), (B) oat spelt xylan (OSX) and (C) wheat arabinoxylan (WAX) (2 mg mL-1) in the presence and absence of regenerated amorphous cellulose (RAC; 2 mg mL-1) before and after incubation with MtLPMO9A (12.5 mg g-1 substrate). Samples were incubated in a 50 mM ammonium acetate buffer (pH 5.0) with ascorbic acid addition (1 mM) or without for 24 h at 52°C. Incubation with MtLPMO9A of all three xylans and xylan-RAC mixtures, in the presence or absence of ascorbic acid, results in the formation of non-oxidized linear xylo-oligomers (XOSn) and substituted xylo-oligomers (black dashed arrow). Incubation of xylan-RAC mixtures with MtLPMO9A in the presence of ascorbic acid results in the formation of non-oxidized gluco-oligomers (GlcOSn) and C1-oxidized gluco-oligomers (GlcOSn#). A + B – The incubation of MtLPMO9A with BiWX-RAC and OSX-RAC mixture in the presence of ascorbic acid results in the formation of numerous products (black arrow), which are not present if MtLPMO9A is incubated with BiWX, OSX or RAC alone. The results of MALDI-TOF MS analysis of BiWX-RAC and OSX-RAC mixture incubated with MtLPMO9A in the presence of ascorbic acid are shown in Figure 4. [file 13068_2015_284_MOESM5_ESM.jpg]

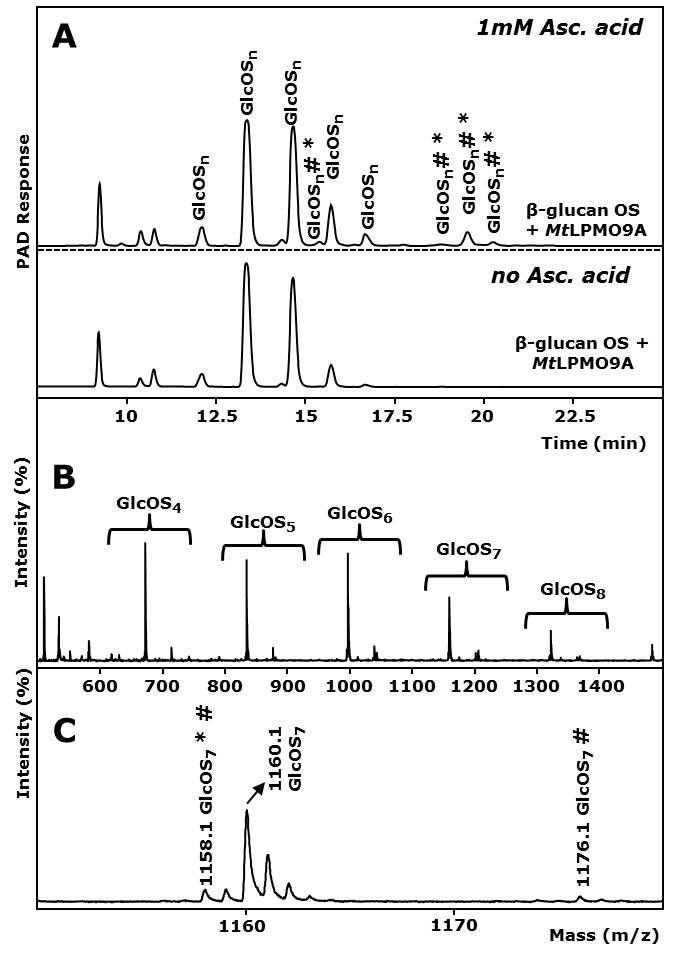

Supplement: Supplementary file 6 — Figure 6. The HPAEC and MALDI-TOF MS analysis of oat spelt (OS) β-glucan incubated with MtLPMO9A. A - HPAEC elution pattern OS β-glucan before and after incubation with partially purified MtLPMO9A fraction SEC-I (Additional Figure 1; 12.5 mg g-1 substrate), with addition of 1 mM ascorbic acid or without. A - In the presence and absence of ascorbic acid, various non-oxidized β-gluco-oligomers (GlcOSn) are formed by the partially purified MtLPMO9A fraction. Incubation of oat spelt β-glucan with MtLPMO9A in the presence of ascorbic acid results in the formation of oxidized gluco-oligomers (GlcOSn#, GlcOSn*). B – MALDI-TOF mass spectrum of partially purified MtLPMO9A incubated with oat spelt β-glucan in the presence of 1 mM ascorbic acid. Clusters of non-oxidized gluco-oligomers and gluco-oligomers, oxidized at C1 (GlcOSn#) and C4 (GlcOSn*) are determined. Enlargement (C) shows the presence of non-oxidized gluco-oligomers and gluco-oligomers, oxidized at C1 with an aldonic acid (#) and at C4 with a keto-group (*). Masses represent lithium (Li) adducts only. [file 13068_2015_284_MOESM6_ESM.jpg]

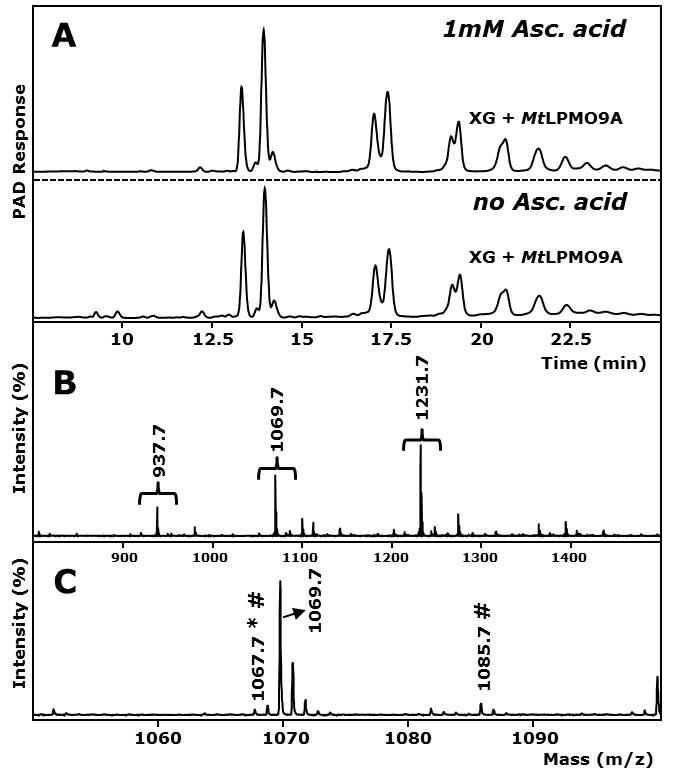

Supplement: Supplementary file 7 — Figure 7. The HPAEC and MALDI-TOF MS analysis of xyloglucan incubated with MtLPMO9A. A - HPAEC elution pattern of xyloglucan from tamarind seed (2 mg mL-1) after incubation with partially purified MtLPMO9A fraction SEC-I (Additional Figure 1; 12.5 mg g-1 substrate). Samples were incubated in 50 mM ammonium acetate buffer (pH 5.0) for 24h at 52°C, either with ascorbic acid addition (1 mM) or without. Numerous various non-oxidized xyloglucan-derived oligomers were formed if xyloglucan was incubated with MtLPMO9A, either with ascorbic acid addition (1 mM) or without. B - MALDI-TOF mass spectrum of xyloglucan incubated with MtLPMO9A with 1 mM ascorbic acid addition. Clusters of non-oxidized and oxidized xyloglucan-derived oligomers are formed. C (enlargement of B) - In the presence of MtLPMO9A and ascorbic acid, next to non-oxidized xyloglucan-derived oligomers, oligomers oxidized at the C1 (#) and C4 (*) position are formed. Masses represent lithium (Li) adducts only. [file 13068_2015_284_MOESM7_ESM.jpg]

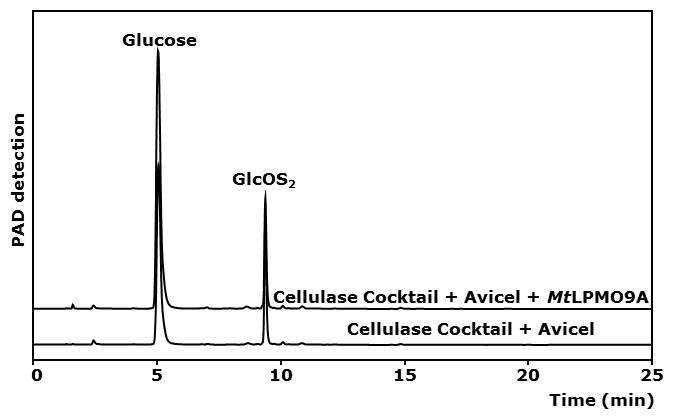

Supplement: Supplementary file 8 — Figure 8. MtLPMO9A incubation with a cellulase cocktail. HPAEC elution patterns of Avicel incubations with a cellulase cocktail (Dyadic, Wageningen, The Nederlands) with and without partially purified MtLPMO9A addition (2.5 mg protein g-1 Avicel). The addition of MtLPMO9A to a cellulase cocktail (5 mg protein g-1 Avicel) results in a 60% higher release of glucose (based on HPAEC-area) compared to the glucose release from Avicel by the cellulase cocktail alone. Samples were incubated in 50 mM acetate buffer (pH 5.0) at 52°C with ascorbic acid addition (1 mM). [file 13068_2015_284_MOESM8_ESM.jpg]
